# Bronchiole adenoma/pulmonary ciliated mucinous nodular papillary tumor: Case series and literature review

**DOI:** 10.1097/MD.0000000000036559

**Published:** 2023-12-15

**Authors:** Shanshan Liu, Xiaoshan Cai, Jianliang Pan, Shaoyun Liu, Jianjun Lin, Xianwen Yue

**Affiliations:** a Department of Cardiac Intensive Care and Rehabilitation, Weifang People’s Hospital, Shandong Province, China; b Department of Pathology, Weifang No. 2 People’s Hospital, Shandong Province, China; c Department of Critical Medicine, Weifang No. 2 People’s Hospital, Shandong Province, China; d Department of Radiology, Weifang Yuandu Hospital, Shandong Province, China; e Department of Chest Surgery, Weifang No. 2 People’s Hospital, Shandong Province, China; f Department of Radiology, Weifang No. 2 People’s Hospital, Shandong Province, China.

**Keywords:** adenomas, bronchiolar adenomas/pulmonary ciliary mucinous nodular papillary tumors, diagnosis and differentiation, immunohistochemistry, lung neoplasms

## Abstract

**Objective:**

To analyze the clinical-pathological characteristics of 3 cases of bronchiolar adenoma/pulmonary ciliary mucinous nodular papillary tumors, and to improve the understanding of bronchiolar adenoma (BA)/ciliated muconodular papillary tumors (CMPT) (bronchiolar adenoma/ciliated muconodular papillary tumor).

**Methods:**

Retrospective analysis was done on the clinical information, diagnosis, and treatment of 3 instances of BA/CMPT at the Second People’s Hospital of Weifang City. By scanning the CNKI, Wanfang, VIP database, and Pubmed database using the English key words “bronchiolar adenoma, ciliated muconodular papillary tumor,” respectively patients with comprehensive clinical data were gathered, and studies from January 2002 to August 2021 that were relevant to the patients were examined.

**Results:**

A total of 35 articles and 71 instances were found, including 3 cases in our hospital, for a total of 74 cases. There were 31 males and 43 females among them, ranging in age from 18 to 84 years (average 63 years), and 15 cases had a smoking history. The majority of them were discovered by physical examination and had no clinical symptoms. The majority of the imaging revealed solid nodules with variable forms, with some ground-glass nodules displaying vacuole and bronchial inflation signs. BA/CMPT are generally gray-white, gray-brown solid nodules with obvious boundaries but no envelope with a maximum dimension of 4 to 45 mm (average 10.6 mm) on gross examination. Acinar, papillary, and lepidic formations can be seen under the microscope at high magnification; the majority of these structures are made up of tripartite epithelial components, including basal cells, mucous cells, ciliated columnar cells, and alveolar epithelial cells, demonstrating a variety of combinations. An important basis for diagnosis in immunohistochemistry is the continuous positive basal cell layer that is shown by p63, p40, and CK5/6. BRAF and epidermal growth factor receptor are the genes that are most frequently mutated. All of the patients showed no signs of metastasis or recurrence during follow-up period.

**Conclusion:**

BA/CMPT is a rare benign tumor of lung epithelium. Because imaging and intraoperative cryosection diagnosis are easy to be misdiagnosed as malignant, it is necessary to further improve understanding and improve immunohistochemistry and genetic examination.

## 1. Introduction

Bronchiolar adenoma (BA) is a new diagnostic word^[1]^ for a benign tumor arising from the bronchiolar mucosal epithelium. The tumor is well-defined yet unenveloped, with a bilayer of cellular structure formed of basal and luminal surface cells as the most notable histological feature. Mucous cells, ciliated cells, Clara cells, and alveolar cells make up the luminal surface cell layer. BA is classified as proximal (proximal type) or distal (distal type) based on the proportion of mucinous cells and ciliated cells. Chang et al^[2]^ introduced this hypothesis in 2018. The name derives from a thorough examination of papillary tumors (Ciliated Muconodular Papillary Tumors, CMPT), which are classified as both typical and nontypical expansions of CMPT. Ishikawa et al,^[3]^ a Japanese scholar, initially described that CMPT, which is a benign or low-grade malignant papillary tumor that occurs in peripheral lung tissue, in 2002. It is named after its morphological traits and is composed of ciliated columnar cells, goblet cells, and basal cells. According to the classical description of CMPT, a subset of proximal-type BAs contain significant papillary features. BA/CMPT was defined as a subtype of epithelial tumor adenoma in the fifth edition of WHO lung tumor classification in 2021, clarifying the link between the 2. This paper retrospectively analyzed 3 confirmed cases of BA/CMPT in Weifang Second People’s Hospital, and summarized and analyzed the related cases with complete data at home and abroad, aiming to improve the understanding of BA/CMPT, help the clinical accurate treatment and evaluation of the disease, and avoid the burden brought by overdiagnosis and treatment to patients.

## 2. Clinical data

### 2.1. Case 1

**Figure 1.**
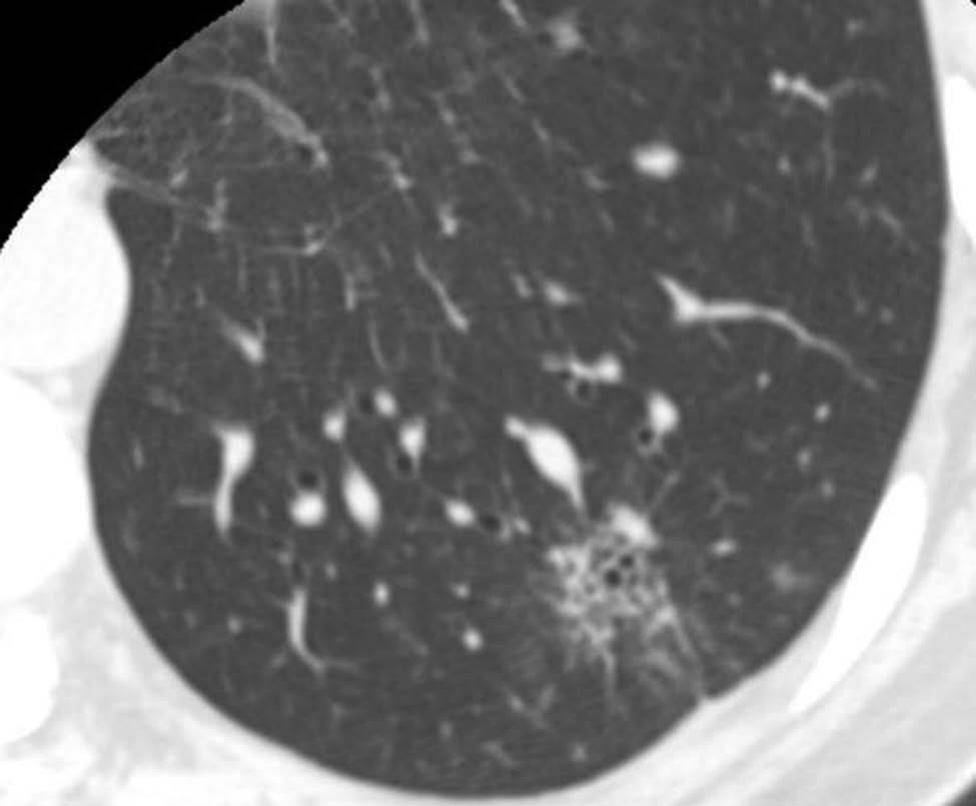
Female, aged 49 years old. Chest CT showed: mixed ground glass nodules in the outer basal segment of the lower lobe of the left lung, with the maximum section diameter of about 14 mm * 14 mm, irregular morphology, unclear boundary, marginal lobation, uneven lesion density, tortuous blood vessels and inflatable bronchi, adjacent pleural adhesion.

**Figure 2.**
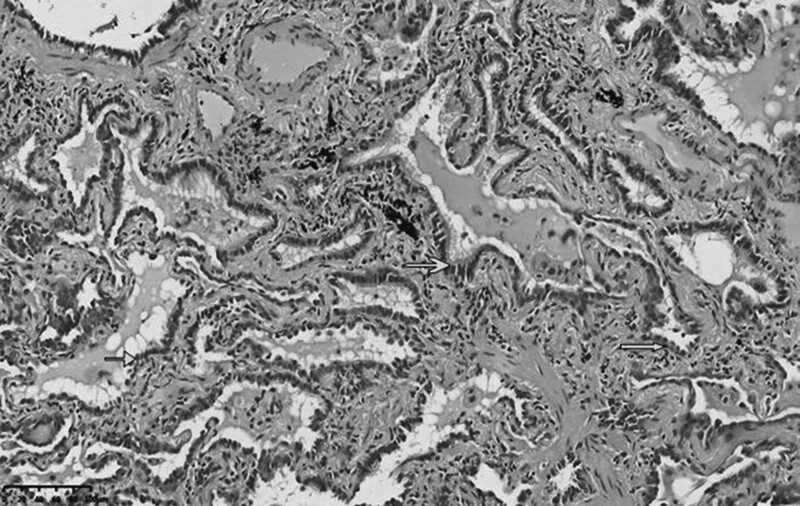
Ciliated cells (long thick arrows), mucous cells (long thin arrow) and basal cells (short thin arrow) are visible in tumors. HE staining for ×200.

**Figure 3.**
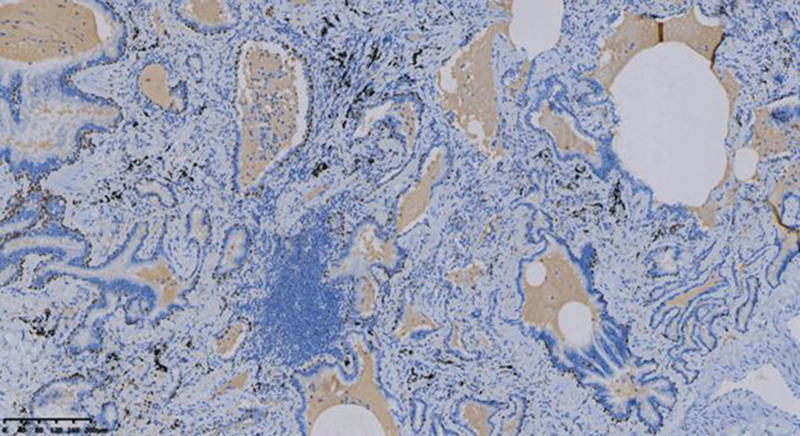
ImmunoHC P40 shows positive nuclear staining of consecutive basal cells. ×200.

**Figure 4.**
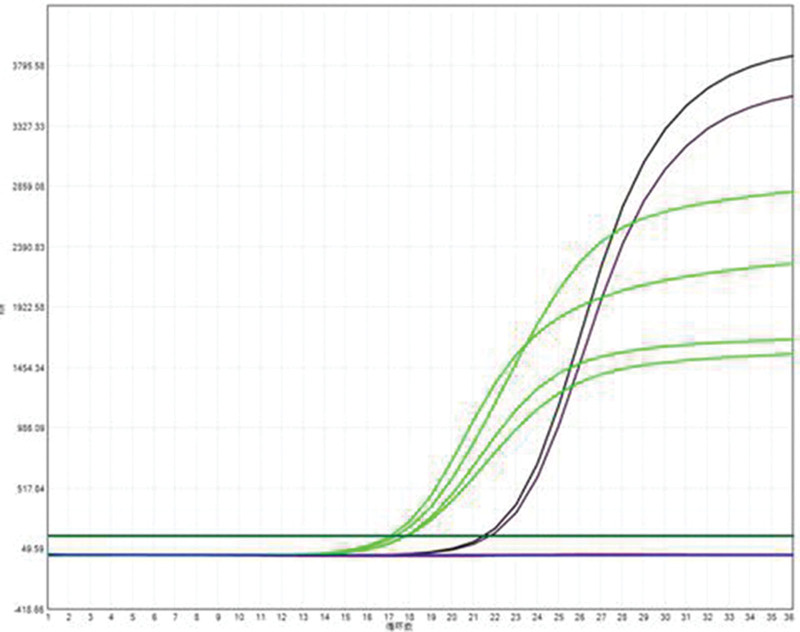
No processes were detected by genetic testing. Quantitative PCR with ARMS fluorescence.

Miss Liu, female, 49 years old. The history of hyperthyroidism is more than 10 years, and the abdominal teratoma resection is more than 10 years. No history of smoking. The patient had no conscious symptoms, and chest CT examination 1 month ago showed: mixed ground glass nodules in the outer basal segment of the left lung (axial maximum section diameter line: 14 mm * 14 mm), irregular morphology, unclear boundary, marginal segmentation, uneven thickened blood vessels and inflatable bronchi, and adjacent pleural adhesion (Fig. 1). One-month review of the chest CT showed no changes. First diagnosis: early lung cancer, hence wedge resection of the lower lobe of the left lung. Gross specimen: gray, gray, brown solid lesions in the open lung, adjacent to the lung surface, no shrinkage in the pleura, and the volume was about 22 mm × 13 mm × 10 mm. Rapid pathology: the dilated adenoid structure in the lesion showed no obvious signs of malignancy. HE staining: ciliated cells, mucus cells, and basal cells were seen in the lesion, and dilated bronchioles were seen around the lesion (Fig. 2). Immunohistochemistry: TTF-1 (+), Napsin A (partial +), CK5/ 6 (basal cell +), P40 (basal cell +), CK (+), P63 (basal cell +), and Ki-67 (<1%) (Fig. 3). Genetic test: no mutated gene was seen (Fig. 4). Pathological diagnosis: bronchiolar adenoma.

### 2.2. Case 2

**Figure 5.**
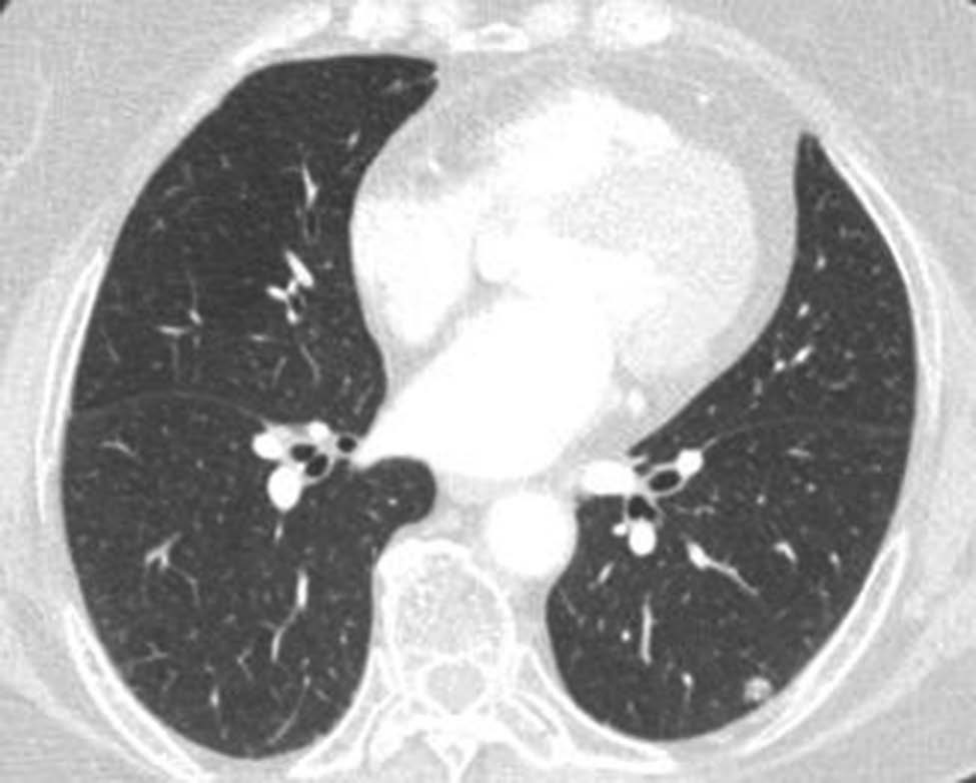
Female, aged 75 years old. Chest CT showed: mixed ground glass nodules in the outer basement segment of the lower lobe of the left lung, the maximum section diameter was about 7 mm * 7 mm, circular shape, clear boundary and marginal segmentation.

**Figure 6.**
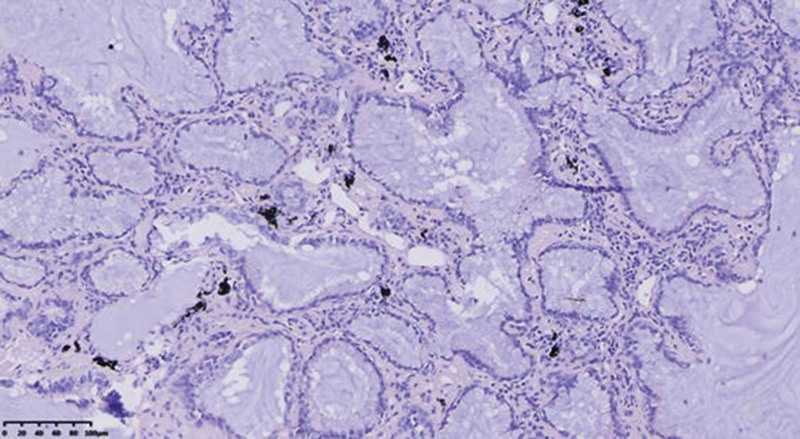
Tumor cells are arranged in acini, composed of mucus cells, large amounts of mucus accumulate in the gland lumen, and basal cells can be distinguished in some areas. HE staining for ×200.

**Figure 7.**
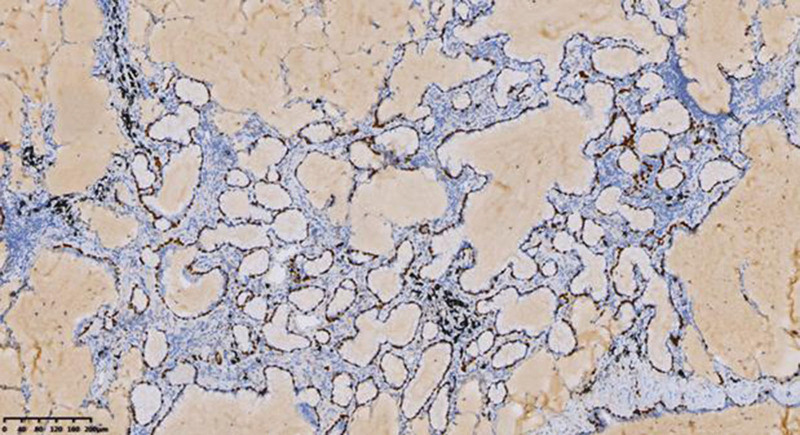
ImmunoHC P63 shows positive nuclear staining of consecutive basal cells. ×100.

**Figure 8.**
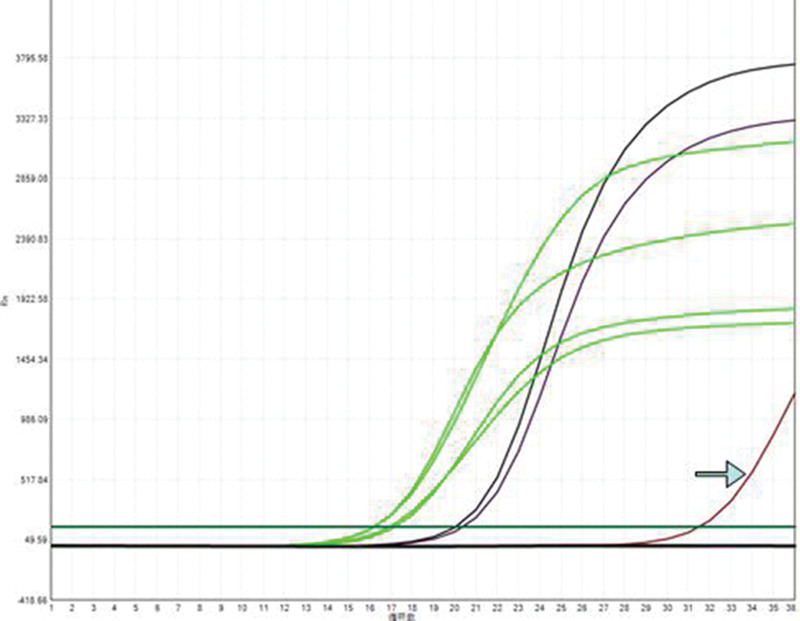
Gene testing shows the BRAF V600E mutation. Quantitative PCR with ARMS fluorescence.

Miss Liu, female, 75 years old. Rectal surgery in 1984 and cholecystectomy in 2009 with no smoking history. The patient had no conscious symptoms. Chest CT examination 1 year ago showed multiple nodules in both lungs, the largest mixed ground glass nodules in the outer basal segment of the left lower lobe (the largest maximum axial section diameter: about 7 mm * 7 mm), circular shape, clear boundary, marginal lobulation, uneven density of lesions, and the solid proportion was more than 50% (Fig. 5).Repeat chest CT 1 year after without change. First diagnosis: early lung cancer, hence wedge resection of the lower lobe of the left lung. Gross specimen: see gray, white, gray and yellow solid lesions in the open lung, adjacent to the lung surface, no pleura shrinkage, and the volume was about 5 mm × 4 mm × 4 mm. Rapid pathology: microscopic lesions showed irregular glands of varying sizes, large amounts of mucus in the gland lumen and surrounding normal alveolar lumen, glandular epithelium was like a single layer of epithelium, and no obvious basal cells, so mucinous adenocarcinoma was considered. HE staining: it is mainly composed of mucous cells, with a small nucleus located at the base of cells, basal cells visible in some areas, and large amounts of mucus accumulation in the glandular lumen (Fig. 6). Immunohistochemistry: TTF-1 (+), Napsin A (foci+), CK5/ 6 (basal cell+), P40 (basal cell+), CK7 (+), P63 (basal cell+), and Ki-67 (<1%) (Fig. 7). Genetic testing: the BRAF V600E mutation (Fig. 8). Pathological diagnosis: bronchiolar adenoma.

### 2.3. Case 3

**Figure 9.**
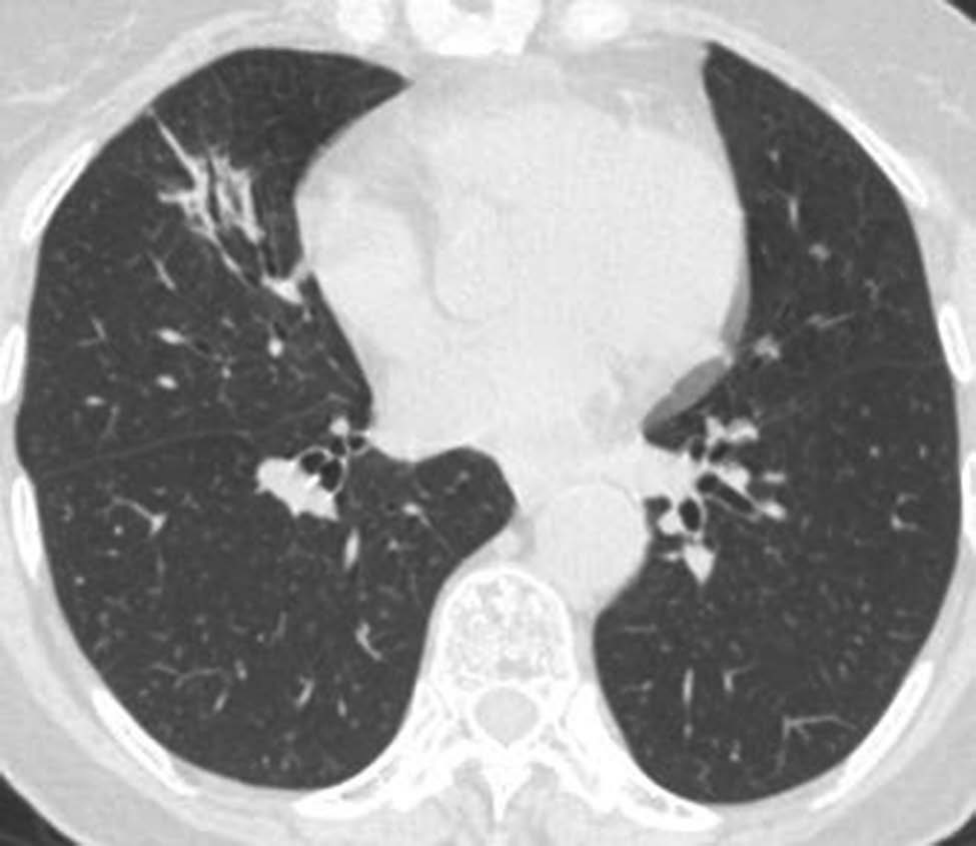
Female, 65 years old. Chest CT showed mixed ground glass nodules in the middle lobe of the right lung, and the maximum section diameter was about 24 mm * 21 mm, circular, clear boundary, marginal lobation, uneven lesion density, tortuous and thickened vessels and inflatable bronchi.

**Figure 10.**
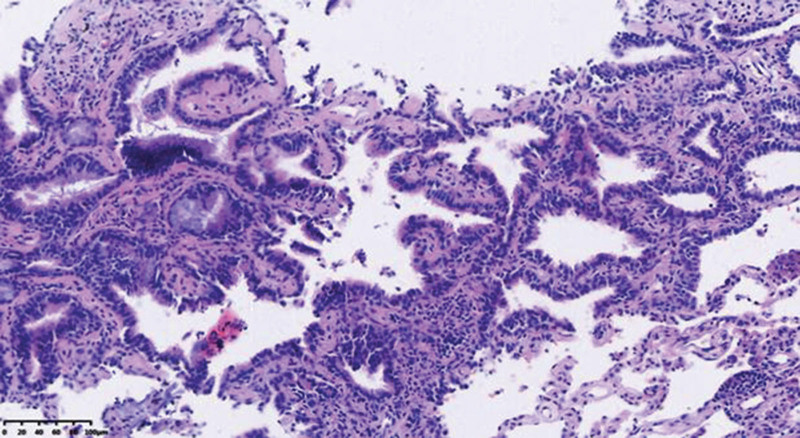
Staining shows that the tumor cells are adherent or acinar, mainly composed of columnar ciliated cells with fewer mucus cells, and basal cells should not be distinguished in conventional sections. HE staining for ×200.

**Figure 11.**
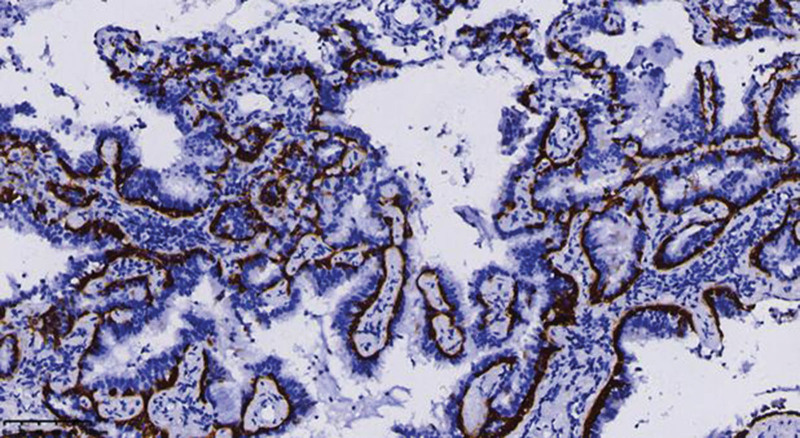
IHC CK5/6 shows cytoplasmic positive coloration of consecutive basal cells. ×200.

**Figure 12.**
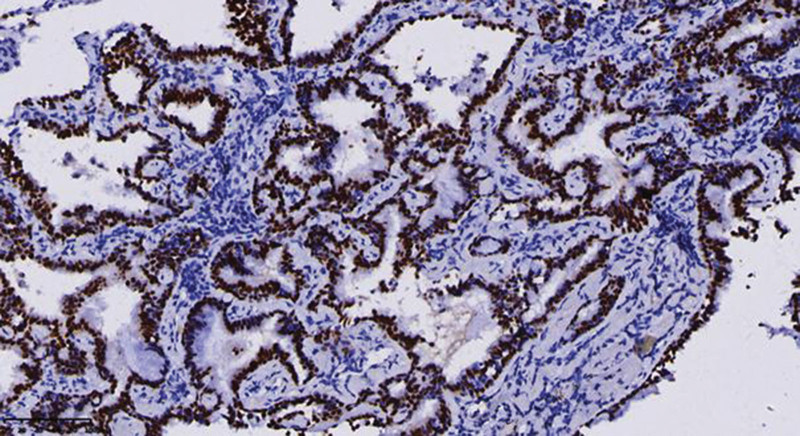
Immunohistochemical TTF-1 showed positive nuclear expression in tumor cells. ×200.

**Figure 13.**
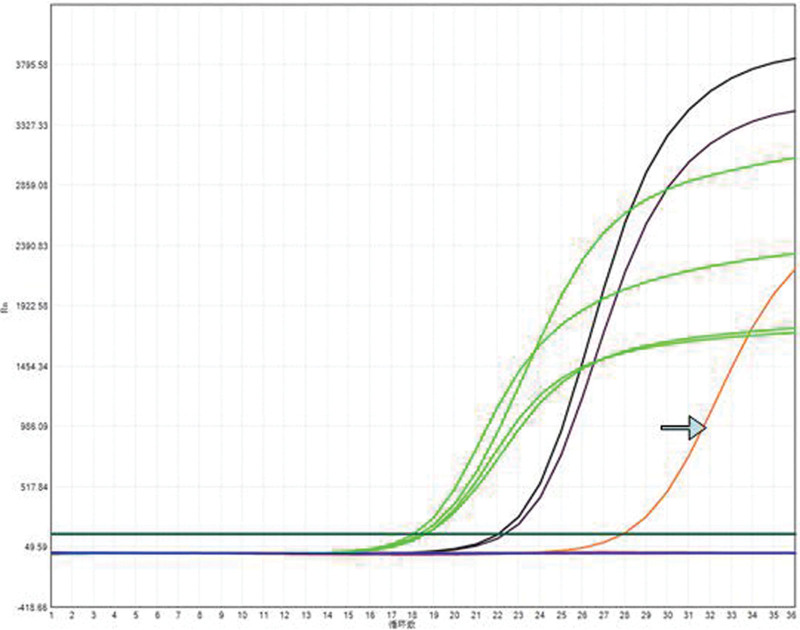
Genetic testing revealed the EGFR 19-del mutation. Quantitative PCR with ARMS fluorescence.

Miss Zhang, female, 65 years old. Appeal of intermittent fever before 1 month, afternoon, the highest 38 ºC, accompanied by pharyngeal discomfort, peripheral body weakness. In the local county hospital, chest CT examination showed: mixed ground glass lesions in the middle lobe of the right lung (maximum axial section diameter: about 24 mm * 21 mm), circular shape, clear boundary, marginal lobation, uneven lesion density, tortuous and thickened blood vessels and inflated bronchi, and adjacent pleural adhesion (Fig. 9).One month interval to our hospital for review of chest CT without change. First diagnosis: early lung cancer, with middle lobectomy of the right lung. Gross specimen: see gray, white, gray and yellow solid lesions in the open lung, adjacent to the lung surface, the pleura was not wrinkled, and the volume was about 19 mm × 18 mm × 14 mm. HE staining: mainly composed of tubular glands, cells are columnar and cilia are visible, and basal cells are not clearly shown in routine sections (Fig. 10). Immunohistochemistry: TTF-1 (+), CK5/ 6 (basal cell+), P40 (basal cell+), P63 (basal cell−), Ki-67 (<1%) (Figs. 11 and 12). Genetic testing: the EGFR 19-del mutation (Fig. 13). Pathological diagnosis: bronchiolar adenoma.

All patients are explicitly state written informed consent was secured from the legal guardian/next of kin for patients within this specific age group in this study.

### 2.4. Literature review

**Figure 14.**
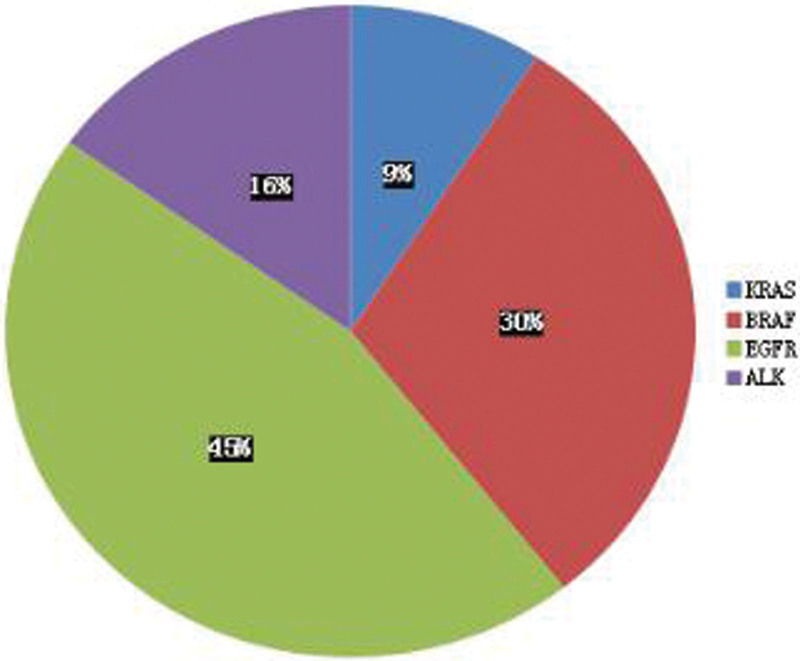
Distribution plots of mutated genes in BA/CMPT.

The literature from January 2002 to August 2021 was searched with “bronchiolar adenoma, pulmonary ciliary mucinous nodule papillary tumor” in CNKI, Wanfang and VIP databases, and 13 Chinese documents were obtained and 29 patients were included. In the PubMed database, “bronchiolar adenoma, ciliated muconodular papillary tumor” and other keywords, the corresponding literature search, obtained 22 English articles, including 42 patients. Most of the documents obtained were case reports, with complete clinical, imaging and pathological data. A total of 35 documents, 71 patients, and 74 cases including 3 cases in our hospital. Clinical data are shown in Table 1: 31 males and 43 females, age range: 18–84 years (mean 63 years);51 cases had no obvious clinical symptoms and 15 cases had smoking history; most of the lesions were located in the right lower lobe, followed by the left lower lobe; tumor size range 4 to 45 mm (mean 10.6 mm), 20 mm accounted for 95.8%; 55 lobe wedge resection, 5 segpectomy and 12 lobectomy; all patients had chest CT images, 45 had solid nodules, 18 had ground glass nodules, 19 nodules were morphologic, 8 had bronchial inflation and 18 had vacuolar features; 5 patients underwent PET/ CT, 2 had low FDG uptake and 3 had no uptake. The pathological results are shown in Table 2 and Figure 14: microscopic adenular, papillary and adherent structures, most basal cells, mucous cells, ciliated cells and alveolar epithelial cells, some tumors with basal cell hyperplasia or scalation, immunohistochemical TTF-1, CK7, p63 positivity, 90.5% of cases Ki67 < 5%, the most mutated gene was BRAF (30.3%) and epidermal growth factor receptor (EGFR) (45.4%).One case underwent postoperative chemotherapy for lung cancer, while other cases did not receive special postoperative treatment. After postoperative follow-up of 1 to 120 months (mean 27 months), all patients showed no metastasis and recurrence during follow-up.

**Table 1:** Clinical characteristics of the BA/ CMPT cases.

Clinical features	Male (n)	Female (n)	Total [n(%)]
*Age of onset (years*) ≤20 21–40	1 0	1 1	2 (2.7) 1 (1.4)
41–60 61–80 >80 *History of smoking* Smoker Nonsmoker *Symptom* Symptomless Have symptoms *Location* Upper lobe of left lung Lobe of left lung Superior lobe of right lung Middle lobe of right lung Inferior lobe of right lung Peripheral type Center type *Size* ≤10 11–20 21–30 >30 *CT representation* Solid nodules Grinding glass nodules *Form* Regulation Irregular *Vacuole sign* Positive Negative *Air bronchogram* Positive Negative *PETCT* FDG no uptake FDG weak uptake FDG strong uptake *Modus operandi* Wedge Pulmonary segmental Resection *Pulmonary lobectomy* After treatment No chemotherapy *Chemotherapy follow-up (m*) ≤20 21–40 41–60 61–80 81–100 >100	8 20 0 14 4 19 2 2 9 5 1 12 24 2 15 11 0 2 21 7 8 19 5 12 4 13 2 2 0 19 3 6 3 1 10 5 4 3 1 0	15 25 3 1 21 32 8 6 11 7 1 20 31 1 31 10 1 0 24 11 11 20 13 16 4 18 1 0 0 36 2 6 13 0 19 6 3 0 0 1	23 (31.1) 45 (60.8) 3 (4.1) 15 (37.5) 25 (62.5) 51 (83.6) 10 (16.4) 8 (10.8) 20 (27.0) 12 (16.2) 2 (2.7) 32 (43.2) 55 (94.8) 3 (5.2) 46 (65.8) 21 (30.0) 1 (14.3) 2 (2.9) 45 (71.4) 18 (28.6) 19 (32.8) 39 (67.2) 18 (39.1) 28 (60.9) 8 (20.5) 31 (79.5) 3 (60.0) 2 (40.0) 0 55 (76.4) 5 (6.9) 12 (16.7) 16 (94.1) 1 (5.9) 29 (55.8) 11 (21.2) 7 (13.5) 3 (5.7) 1 (1.9) 1 (1.9)

BA = bronchiolar adenoma, CMPT = ciliated muconodular papillary tumors, EGFR = epidermal growth factor receptor.

**Table 2:** Pathological characteristics of the BA/CMPT cases.

Pathological features	Male (n)	Female (n)	Total [n(%)]
*Tumor cell* Ciliary columnar cells Basal cells Mucilage cell	28 29 29	32 43 40	60 (29.9) 72 (35.8) 69 (34.3)
*Immunohistochemical* CK7 (+) MUC5AC (+) CEA (+) TTF-1 (+) MUC1 (+) P63 (+) P53 (+) CK20 (+) *Ki-67* 0 <5% ≥5% *Genic mutation* KRAS (+) BRFR (+) EGFR (+) ALK (+)	17 4 11 17 6 15 5 2 2 9 1 2 6 4 2	36 13 11 37 6 32 4 2 2 25 3 1 4 11 3	53 (24.3) 17 (7.8) 22 (10.1) 54 (24.8) 12 (5.5) 47 (21.6) 9 (4.1) 4 (1.8) 4 (9.5) 34 (81.0) 4 (9.5) 3 (9.1) 10 (30.3) 15 (45.4) 5 (15.2)

BA = bronchiolar adenoma, CMPT = ciliated muconodular papillary tumors, EGFR = epidermal growth factor receptor.

## 3. Discussion

BA/CMPT is a rare benign tumor of lung epithelial origin, which was first reported by Japanese scholar Ishikawa^[3]^ in 2002 and named “papillary tumor (ciliated muconodular papillary tumor, CMPT).” In 2018, Chang et al^[2]^ proposed the concept of “bronchiolar adenoma (Bronchiolar Adenoma, BA)” and divided it into 2 subtypes: proximal and distal type, with CMPT belonging to proximal BA. In the fifth edition of WHO thoracic tumor classification in 2021,^[4]^ combined BA and CMPT as a new subtype of lung adenoma, and the ICD-O code was 8140/ 0. According to the collected literature and a total of 74 cases in our hospital, 31 cases were male and 43 female (male to female ratio was about 1:1.7), the age of onset was 18–84 years (mean age 63 years), and more middle-aged and elderly patients (68 cases over 50 years old, 91.89%).Site: right lower lobe (32 cases, 43.2%) > left lower lobe (20 cases, 27.0%) > right upper lobe (12 cases, 16.2%) > left upper lobe (8 cases, 10.8%) > right middle lobe (2 cases, 2.7%). Lesion size range: 4 to 45 mm (mean 10.6 mm), 20 mm accounted for 95.8%. Most of them had no clinical symptoms (51 cases, 83.6%), and chest CT examination was found incidentally, and the clinical symptoms were mostly nonspecific manifestations such as cough and sputum. Of these, 40 had studied smoking history and 25 were nonsmokers.

Most of the literature focuses on the pathology of BA/CMPT, while the chest imaging findings are rarely reported in the literature. All 3 cases in our hospital were ground glass lesions, 2 cases had irregular morphology, 2 cases had clear border, 3 cases had marginal segmentation, 2 cases had inflatable bronchial features in the lesion, and 2 cases showed pleural adhesion but all showed single fine line findings. According to the collected literature and a hospital of 74 cases of chest imaging findings, the following are: 55 cases (94.8%) for peripheral nodules, 45 cases (71.4%) for solid nodules, 18 cases (28.6%) for ground glass nodules, 19 cases (32.8%) of morphological rules, 8 cases (20.5%) with bronchial inflation, 18 cases (39.1%) with vacuolation, bronchial inflation and vacuolation symptoms occurred in more cases of ground glass. Five patients obtained PET/CT scans; 2 had minimal FDG uptake and 3 had no uptake. During imaging follow-up, the majority of cases grew gradually or slowly, with an average yearly growth rate of 0.49 mm/year,^[5]^ and were easily misinterpreted as malignant lung lesions for surgical treatment.

BA/CMP pathological gross examination, usually for a clear single nodules, cut surface gray or gray brown, solid, medium or soft texture, some cases can have microcystic changes, accompanied by mucus secretion can be glue or mucoid, no capsule, mostly around the lung or around the bronchioles, usually do not appear pleural sag.^[6]^ BA/CMP is mainly manifested as glandular cavity or papillary structure, and the lesion is clearly separated from the surrounding normal lung tissue. In some cases, mucus components can be seen in the glandular cavity, and sometimes bronchioles can be seen within or around the lesion. Under high microscopic, 3 epithelial components can be seen in classical lesions: ciliated cells, mucous cells and basal cells. In some cases, ciliated cells and mucous cells are few or absent, with little nuclear atypia, and usually no pathological nucleotic phase and necrosis. The cellular structure of the bilayer in the typical case is obvious, and it is not difficult to diagnose. Sometimes, basal cells are not easy to be found in HE staining, especially in intraoperative frozen sections, which is easy to be confused with adenocarcinoma, and we need to use the immunohistochemical staining markers of basal cells (p40, p63, CK5/6) to identify.^[7]^ Immunohistochemistry showed the continuous presence of basal cells in the gland lumen or around the papilla, which is also the key evidence for the diagnosis of BA/CMPT. Proliferation index (Ki-67 index) is generally <5%.^[8]^ According to the pooled literature and the immunohistochemical results of 74 cases in our hospital: 53 CK7 (+), 17 MUC5AC (+), 22 CEA (+), 54 TTF-1 (+), 12 MUC 1 (+), 47 P63 (+), 9 P53 (+), 4 CK20 (+), most Ki-67 low expression (0–10%), 4 were 0, 34 < 5%, 45%. Also, genetic mutations can be detected in some cases of BA/CMPT. According to the collected literature and the genetic test results of 74 cases, BRAF mutations and EGFR mutations account for the majority, among which BRAF mutations are mostly V600E mutations. The most common type of mutation in EGFR is exon 19 E746_S752delinsV, an extremely rare mutation in EGFR mutant lung adenocarcinoma; KRAS and HRAS mutations are at codons 12 and 13; all AKT 1 mutations are restricted to the E17K mutation. The presence of mutations in the above genes supports that BA/CMPT is a tumor rather than reactive hyperplasia or metaplastic process.^[9]^

Differential diagnosis: ① invasive mucinous adenocarcinoma: in common is both gland cavity structure or nipple structure, mainly have mucus secretion of columnar cells, usually mucinous adenocarcinoma rarely cilia or only a little immature cilia structure, part of the gland cavity filled with mucus, tumor cells is relatively small, atypia is not obvious, but there is no complete basal cells, which is different from BA/CMPPT. ② Highly differentiated mucoepidermoid carcinoma: this kind of tumor can also contain large amounts of mucus components, tumor cells are mainly composed of mucus cells, epidermoid cells and intermediate cells, which occurs in the main bronchus of young people, generally without papillary structure, immunohistochemical marker CD117 positive, and no complete basal cells. ③ Acinar type adenocarcinoma: mainly presented as acinar structure, but no mucus in the cavity, tumor cells have a certain atypia, visible small nucleoli or nuclear division phase. No intact basal cells are present. Distal BA/CMPT can often find a continuity relationship with normal bronchioles. ④ In situ adenocarcinoma: This type of tumor needs to be differentiated from distal BA/CMPT. The cells of both tumors have less atypia and have adherent growth mode, but in situ adenocarcinoma is mainly tubular glands, and the cells are often cubic or low column. It is often misdiagnosed in frozen sections, and the differentiation between the 2 is mainly determined by immunohistochemical labeling of basal cells to confirm.^[10–12]^

BA/CMPT was classified as a benign lung epithelial tumor in the fifth edition of the WHO lung tumor classification, with the majority of cases being solid or ground glass nodules with peripheral spread discovered by chest CT. There was no recurrence or metastasis recorded during follow-up after sublobectomy or lung resection. However, a few CMPT malignancies progressed to mucinous adenocarcinoma^[13]^ and malignant ciliary mucus-nodular papillary tumors^[14]^ were identified. A small number of cases have since been discovered to have partial loss of basal cells; specialists feel that this form of tumor is an atypical bronchiolar adenoma, which can be treated clinically as benign.^[15]^ Because BA/CMPT is a new disease, we must continue to collect cases and undertake more intensive research. The limitations of this study include the limited sample size and the need to further increase and expand the sample size.

## Acknowledgments

This work was supported by the Health Commission of Weifang (WFWSJK-2022-150, WFWSJK-2020-049, WFWSJK-2020-078).

## Author contributions

**Conceptualization:** Xianwen Yue.

**Data curation:** Shanshan Liu, Xiaoshan Cai, Shaoyun Liu.

**Formal analysis:** Xiaoshan Cai.

**Resources:** Shanshan Liu.

**Writing – original draft:** Jianliang Pan.

**Writing – review & editing:** Jianjun Lin.

## References

[1] Wang E. Bronchiolar adenoma: a benign tumor easily confused with carcinoma. Chin J Pathol. 2019:425–32.

[2] Chang JC Montecalvo J Borsu L. Bronchiolar adenoma: expansion of the concept of ciliated muconodular papillary tumors with proposal for revised terminology based on morphologic, immunophenotypic, and genomic analysis of 25 cases. Am J Surg Pathol. 2018;42:1010–26. 29846186 10.1097/PAS.0000000000001086 PMC8063713

[3] Ishikawa Y. Ciliated muconodular papillary tumor of the peripheral lung: benign or malignant? Pathol Clin Med (Byori to Rinsho). 2002;20:964–5.

[4] Tsao M. PL0105 The new WHO classification of lung tumors. J Thorac Oncol. 2021;16:S63.

[5] Kao TH Yeh YC. Ciliated muconodular papillary tumor/bronchiolar adenoma of the lung. Semin Diagn Pathol. 2021;38:62–71. 33985833 10.1053/j.semdp.2021.04.002

[6] Shen L Lin J Ren Z. Ciliated muconodular papillary tumor of the lung: report of two cases and review of the literature. J Surg Case Rep. 2019;2019:rjz247. 31528329 10.1093/jscr/rjz247 PMC6736349

[7] Guo Y Shi Y Tong J. Bronchiolar adenoma: a challenging diagnosis based on frozen sections. Pathol Int. 2020;70:186–8. 31994796 10.1111/pin.12901

[8] Shao K Wang Y Xue Q. Clinicopathological features and prognosis of ciliated muconodular papillary tumor. J Cardiothorac Surg. 2019;14:143. 31340823 10.1186/s13019-019-0962-3 PMC6651997

[9] Yang C Wang X Da J. Distal-type bronchiolar adenoma of the lung harboring an EGFR exon 21 pL858R mutation: a case report. Thorac Cancer. 2020;11:3596–8. 33063939 10.1111/1759-7714.13692 PMC7705926

[10] Abe M Osoegawa A Miyawaki M. Ciliated muconodular papillary tumor of the lung: a case report and literature review. Gen Thorac Cardiovasc Surg. 2020;68:1344–9. 31749068 10.1007/s11748-019-01252-x

[11] Liu S Liu N Xiao M. First case of bronchiolar adenoma lined purely by mucinous luminal cells with molecular analysis: a case report. Medicine (Baltim). 2020;99:e22322. 10.1097/MD.0000000000022322 PMC7523813 32991441

[12] Tachibana M Saito M Kobayashi J. Distal-type bronchiolar adenoma of the lung expressing p16^INK4a^ - morphologic, immunohistochemical, ultrastructural and genomic analysis - report of a case and review of the literature. Pathol Int. 2020;70:179–85. 32030846 10.1111/pin.12904 PMC7079048

[13] Han X Hao J Ding S. Bronchiolar adenoma transforming to invasive mucinous adenocarcinoma: a case report. Onco Targets Ther. 2021;14:2241–6. 33833523 10.2147/OTT.S299864 PMC8019666

[14] Wang F Shen MH Cao D. Malignant ciliated muconodular papillary tumors of the lung: a case report. Int J Surg Pathol. 2021;29:520–3. 33605184 10.1177/1066896920988359

[15] Zhang J Shao JC Han YC. [Issues on pathological diagnosis of bronchiolar adenoma]. Zhonghua Bing Li Xue Za Zhi. 2020;49:529–33. 32486526 10.3760/cma.j.cn112151-20190821-00459

